# Reply: Diabetogenic Potential of Ancestral and Modern Wheat Landraces, *Nutrients* 2017, *9*, 816

**DOI:** 10.3390/nu9090922

**Published:** 2017-08-23

**Authors:** Jonathan Gorelick, Ludmila Yarmolinsky, Arie Budovsky, Boris Khalfin, Joshua D. Klein, Yosi Pinchasov, Maxim A. Bushuev, Tatiana Rudchenko, Shimon Ben-Shabat

**Affiliations:** 1 Eastern Regional Research and Development Center, Judea Center, Kiryat Arba 90100, Israel; yludmila@post.bgu.ac.il (L.Y.); abudovsky@gmail.com (A.B.); 2 Department of Clinical Biochemistry and Pharmacology, Ben-Gurion University of the Negev, Beer Sheva 84990, Israel; boriskh83@gmail.com (B.K.); sbs@bgu.ac.il (S.B.-S.); 3 Institute of Plant Sciences, ARO-Volcani Center, Rishon LeZion 50250, Israel; vcjosh@volcani.agri.gov.il; 4 Siap Laboratory, Rehovot 76267, Israel; siap.013.lab@gmail.com; 5 Department of Information Science and Systems, Morgan State University; Baltimore, MD 21251, USA; maxim.bushuev@morgan.edu; 6 Scheller College of Business at Georgia Tech, Atlanta, GA 30308, USA; tatiana.rudchenko@scheller.gatech.edu

Our “diabetogenic diet” composition [1] was indeed based on the one described by Funda et al. [2] with regards to dietary requirements. However, as correctly pointed out, the ingredients utilized to reach the final nutritional levels were somewhat different (See Table 1 [1] for a list of each diet’s composition). As mentioned [1], these differences, specifically the increased percentage of maize and tomato fibers, may at least partially explain the surprising observation of an increased incidence of diabetes in Non-Obese Diabetic (NOD) mice on a non-wheat diet. However, further research is needed to confirm this hypothesis.

Regarding the wheat utilized in the study, whole wheat grains were used for all wheat sources tested including conventional wheat bread grains. As mentioned [1], “harvested seeds were analyzed for nutritional content and were milled to produce the diets for the animal study.” No proteins were isolated nor was anything other than whole wheat grains utilized to produce the flour incorporated into the experimental diets.

Regarding the diagnosis of diabetes, as stated [1], none of the mice in groups 3, 4, or 5 developed diabetes. The deviations observed were not due to mice with glucose levels above 130. The highest levels observed in groups 3, 4, or 5 was around 120 mg/dL in a few outlying mice. There were, however, a number of mice with lower glucose levels, increasing the standard deviation. Overall, the difference between groups 1–2 and 3–5 was statistically significant as stated.

As stated in the “Experimental design”, 10 animals were used for each diet. All analyses including glucose, insulin, cholesterol, interferon-γ (IFN-γ), and IL-10 levels utilized all of the animals. No subsamples were used in this research. We hope we have clarified all of the issues raised.
